# Integrated Metabolomics and Network Pharmacology Approach to Explain Possible Action Mechanisms of Xin-Sheng-Hua Granule for Treating Anemia

**DOI:** 10.3389/fphar.2018.00165

**Published:** 2018-03-02

**Authors:** Han-Qing Pang, Shi-Jun Yue, Yu-Ping Tang, Yan-Yan Chen, Ya-Jie Tan, Yu-Jie Cao, Xu-Qin Shi, Gui-Sheng Zhou, An Kang, Sheng-Liang Huang, Ya-Jun Shi, Jing Sun, Zhi-Shu Tang, Jin-Ao Duan

**Affiliations:** ^1^College of Pharmacy and Shaanxi Collaborative Innovation Center of Chinese Medicinal Resources Industrialization, Shaanxi University of Chinese Medicine, Xianyang, China; ^2^Jiangsu Collaborative Innovation Center of Chinese Medicinal Resources Industrialization, Nanjing University of Chinese Medicine, Nanjing, China; ^3^Jiangsu Key Laboratory for High Technology Research of TCM Formulae, National and Local Collaborative Engineering Center of Chinese Medicinal Resources Industrialization and Formulae Innovative Medicine, Nanjing University of Chinese Medicine, Nanjing, China; ^4^Jiangsu Revolence Pharmaceutical Co., Ltd., Huai’an, China

**Keywords:** Xin-Sheng-Hua granule, anemia, metabolomics, network pharmacology, molecular mechanism

## Abstract

As a well-known traditional Chinese medicine (TCM) prescription, Xin-Sheng-Hua Granule (XSHG) has been applied in China for more than 30 years to treat postpartum diseases, especially anemia. However, underlying therapeutic mechanisms of XSHG for anemia were still unclear. In this study, plasma metabolomics profiling with UHPLC-QTOF/MS and multivariate data method was firstly analyzed to discover the potential regulation mechanisms of XSHG on anemia rats induced by bleeding from the orbit. Afterward, the compound-target-pathway network of XSHG was constructed by the use of network pharmacology, thus anemia-relevant signaling pathways were dissected. Finally, the crucial targets in the shared pathways of metabolomics and network pharmacology were experimentally validated by ELISA and Western Blot analysis. The results showed that XSHG could exert excellent effects on anemia probably through regulating coenzyme A biosynthesis, sphingolipids metabolism and HIF-1α pathways, which was reflected by the increased levels of EPOR, F2, COASY, as well as the reduced protein expression of HIF-1α, SPHK1, and S1PR1. Our work successfully explained the polypharmcological mechanisms underlying the efficiency of XSHG on treating anemia, and meanwhile, it probed into the potential treatment strategies for anemia from TCM prescription.

## Introduction

Anemia, one of the most common diseases in obstetrics, could cause severe maternal and fetal complications, including preterm birth and placental mesenchymal dysplasia (Mousa et al., 2014). On a global scale, more than 500,000 maternal deaths occurred during pregnancy each year, and 20% of them are resulted from anemia and postpartum hemorrhage (Balarajan et al., 2011; Milman, 2011). Researchers have demonstrated that postpartum anemia was commonly caused by blood clots, coagulation abnormalities, retained placenta or genital tract trauma (Froessler et al., 2014; Markova et al., 2015). However, the available therapeutic drugs and their therapeutic effects were limited, and some of them even caused obvious side effects (Milman, 2012; Tunçalp et al., 2013). For these reasons, the discoveries of potential strategies for treating anemia could have a positive effect on maternal as well as fetal outcomes.

Traditional Chinese medicine (TCM) prescriptions are usually comprised of some specific herbs in order to achieve the synergistic effects in a holistic way (Yang et al., 2017). Comparing with modern medicine, they had obvious advantages of fewer side effects for treating multi-factorial diseases (Wang et al., 2016b). In the past few decades, Xin-Sheng-Hua granule (XSHG) has been widely applied for the treatment of anemia-related diseases in China, especially for the anemia caused by postpartum hemorrhage, whose prescription is the boiled water extraction followed by seven common herbs, *Angelica sinensis* (Oliv.) Diels (ASD; Danggui), *Leonurus artemisia* (Laur.) S. Y. Hu F (LA; Yimucao), *Ligusticum chuanxiong* Hort. (LC; Chuanxiong), *Prunus persica* (L.) Batsch (PP; Taoren), roasted rhizome of *Zingiber officinale* Rosc. (RZR; Jiangtan), Radix *Glycyrrhizae preparata* (RGP; Zhigancao) and *Carthamus tinctorius* L. (CT; Honghua), at a proportion of 80:90:30:8:5:5:5 (Pang et al., 2016b). According to the National Bureau of Statistics of China^1^, its annual sales are expected to exceed 100 million dollars by 2020, and it ranked the forefront among all of the obstetrical medications. A growing body of evidence indicated that XSHG could exhibit excellent therapeutic effects on anemia via promoting hematopoietic stem cell proliferation and differentiation (Pang et al., 2017; Zhou et al., 2017). However, up to date, the action mechanism for treating anemia of XSHG remained poorly understood.

Metabolomics aims at constructing the metabolic profile of endogenous metabolites with low molecular weight in biological systems through modern analytical techniques (Naz et al., 2014). It could be applied to monitor and diagnose disease progression by evaluating the content variations of metabolic biomarkers between control and model groups (Courant et al., 2014). Due to these advantages, increasing attention has been focused on metabolomics to reveal the mechanisms of TCM prescriptions, such as Huangqin Decoction (Cui et al., 2017) and Liu Wei Di Huang Wan (Wang et al., 2010). Network pharmacology is now regarded as a holistic and efficient technique to study the role of TCM prescriptions (Yue et al., 2017b). It can help to understand the active compounds and therapeutic targets of TCMs by combining pharmacology and pharmacodynamics analysis (Wang et al., 2017). Thus this approach is quite important for interpreting the underlying mechanisms of TCMs (Chen et al., 2016; Guo et al., 2016). Recently, some researchers have successfully used the integrated metabolomics and network pharmacology strategy to explore the interactions between organisms and drugs (Kell and Goodacre, 2014; Li S. et al., 2014), bringing great inspiration to the mechanism research of XSHG.

**FIGURE 1:**
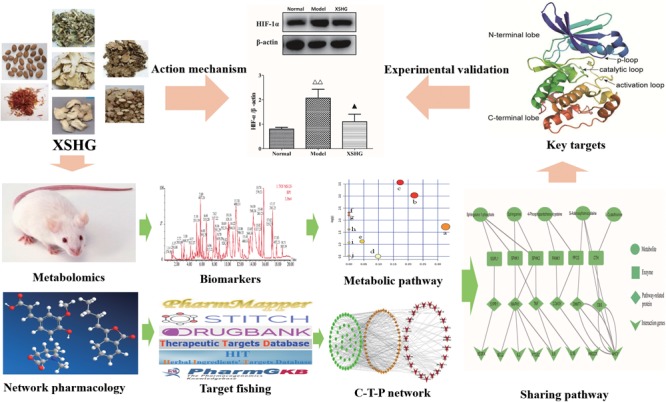
The integrated strategy of metabolomics and network pharmacology. C-T-P network: compound-target-pathway network.

In current study, in order to explain possible action mechanisms of XSHG for treating anemia, we performed the integrated strategy of metabolomics and network pharmacology (**Figure 1**). Firstly, the anemia model was induced by bleeding from the orbit. Secondly, the metabolic pathways were performed by studying plasma metabolomics through ultra-high-performance liquid chromatography combined with quadrupole time-of-flight mass spectrometry (UHPLC-QTOF/MS) and multivariate data method. Thirdly, the anemia-relevant pathways were profiled and potential mechanisms of XSHG were revealed through network pharmacology. Finally, the crucial targets of the shared pathways of metabolomics and network pharmacology were experimentally validated to account for the therapeutic effects of XSHG. This approach could offer a significant guidance for elucidating mechanism of XSHG on anemia, and then promote its clinical application.

## Materials and Methods

### Chemicals and Reagents

The prescription XSHG was generously provided by Jiangsu Revolence Pharmaceutical Co., Ltd. (Huai’an, China). Acetonitrile, methanol and formic acid of LC-MS grade were purchased from Merck KGaA (Darmstadt, Germany). Ethylene diamine tetraacetic acid-2Na (EDTA-2Na) was offered by Shanghai Danning Chemical Co., Ltd. (Shanghai China). The deionized water purified by Milli-Q water purification system (Millipore, Billerica, MA, United States) was applied to prepare and extract plasma samples. Other reagents and chemicals were all analytical grade.

### Animals and Drug Administration

Sprague-Dawley (SD) female rats (220 - 250 g) were purchased from Shanghai Slac Laboratory Animal Co., Ltd., (Shanghai, China). Before the experiments, the animals were housed in an environmentally controlled breeding room for 12 h light-dark cycle, at a relative humidity of 55 ± 5°C and a temperature for 20 ± 2°C. All experimental animals were free access to food and tab water during this study. The protocol was strictly in accordance with the Guide for the Care and Use of Laboratory Animals (US National Research Council, 1996), and was approved by the Animal Experimental Ethics Committee of Nanjing University of Chinese Medicine.

After 10 days for acclimatization, 30 SD rats were randomly divided into 3 groups with 10 rats in each: the control, model and XSHG groups. The method of inducing anemia model was according to our previous research (Shi et al., 2014a). Except the control group, rats in other groups were performed by bleeding from the orbit at the account of 5.0 mL/kg each day, and the experimental period was 12 successive days.

The XSHG extract was dispersed and dissolved homogeneously by using pure water, and its dosage of 9.86 g/kg in XSHG group was equivalent to two times of the adult daily dose of its prescription (36 g), which was inferred from the following formula: human dose of crude herbs in clinic × 0.018/200 × 1000 × the multiple of clinical equivalency dose (Shi et al., 2014b). Model and control groups were intragastrically administered with the same volume of saline solution, and all animals were given orally one time each day for 12 days. After 1 h of the last administration, 1.0 mL blood samples were collected into 1.5 mL centrifuge tubes via the orbit. 200 μL whole blood was applied to evaluate peripheral blood routine, including hemoglobin (HGB/g.L^-1^), red blood cell count (RBC/10^12^.L^-1^), white blood cell count (WBC/10^9^.L^-1^), platelet count (PLT/10^9^.L^-1^), lymphocyte ratio (LY/%), monocyte ratio (MO/%), neutrophils ratio (GR/%) and haematocrit (HCT/%). The rest of whole blood samples were immediately centrifuged at 3000 rpm for 10 min, then the supernatant was transferred into another 1.5 mL centrifuge tube. Tissue samples from right lobe of liver were grinded with saline solution (1:3, v/v). All of the plasma and liver samples were kept at -80°C until analysis.

### Metabolomics Study

### Sample Preparation

Plasma samples were thawed at room temperature before preparation. To precipitate protein, 200 μL plasma samples were extracted with 600 μL methanol, and the mixture was then vortexed for 1 min and centrifuged at 3000 rpm for 10 min. Afterward, 400 μL supernatants were transferred into new centrifuge tubes, and were evaporated to dryness at 37°C by using Labconco CentriVap concentrator (Kansas City, MO, United States). The residues were re-dissolved with 100 μL of initial mobile phase, vortexed for 3 min and centrifuged at 13000 rpm for 10 min at 4°C. Finally, an aliquot of 5 μL supernatant was injected for UHPLC-MS analysis.

Furthermore, 15 plasma samples were randomly selected from these three groups, which were mixed together as quality control (QC) samples. As QC samples contained the most data of each group, it was applied to validate the stability of UHPLC-MS system. Before analysis, QC samples were analyzed for six times to equilibrate the UHPLC system, then every ten samples were used to monitor the stability of this method (Godzien et al., 2011; Prosser et al., 2014). All samples above were maintained at 4°C during analysis.

#### Liquid Chromatography

Chromatographic separation was performed on an UHPLC^TM^ system (Waters, Corp., Milford, MA, United States). Considering the complicated analytes, a Thermo Scientific Hypersil GOLD column (100 mm × 3 mm, 1.9 μm) was firstly applied for all the analytes. And the column temperature was maintained at 35°C. Chromatographic analysis was performed with gradient elution using water/0.1% formic acid (solvent A) and acetonitrile (solvent B), which programmed as follows: 5–40% B from 0 to 3 min; 40–52% B from 3 to 3.5 min; 52% B from 3.5 to 6 min; 52–95% B from 6 to 17 min; 95% B from 17 to 19 min; 5% B from 19 to 19.5 min, held at 5% B for 3 min to equilibrate the column. The mobile phase was directly delivered into electrospray ion (ESI) source at 0.4 mL/min.

#### Mass Spectrometry

In the experiment, a Waters Synapt^TM^ QTOF/MS spectrometer (Waters Corp., Milford, MA, United States) was combined with UHPLC system via the ESI source in both positive and negative ionization modes. The detailed parameters were as follows: extraction cone voltage of 2.0 V, sample cone voltage of 30.0 V, capillary voltage of 3.0 kV, desolvation temperature of 400°C, and source temperature of 120°C. Nitrogen was used as the cone and desolvation gas at the flow rate of 50 and 800 L/h accordingly. The metabolomics in centroid mode were set from 100 to 1000 Da. To ensure accuracy, leucine encephalin was selected as lock-mass solution for the acquisition of accurate mass.

#### Pattern Recognition Analysis and Data Processing

The acquired raw data were introduced to MakerLynx within MassLynx software for peak alignment and detection, then m/z data and retention time of each analyte could be obtained. The primary parameters were: mass range 100–1000 amu, retention time range 1–20 min, mass tolerance 0.1 Da, and noise elimination level 5. After normalization, the resultant data matrices were imported to EZinfo 2.0 for principal component analysis (PCA), partial least-squares-discriminant analysis (PLS-DA), and orthogonal projection to latent structures (OPLS) analysis (Ciborowski et al., 2012). Prior to multivariate statistical analysis, raw data acquired from UHPLC–MS were scaled to Pareto variance. In PLS-DA score plots, the variables with VIP > 1 were usually regarded as candidate biomarkers and were subjected to further identification of the chemical formula. Pathway analysis was conducted with MetaboAnalyst, a web-based tool for visualization of metabolomics results (Park et al., 2015; Takayama et al., 2015).

### Network Pharmacology Study

#### Compound Data Preparation and ADME Screening

All of the compounds data of seven herbs in XSHG were obtained from traditional Chinese medicine systems pharmacology database and analysis platform,^2^ (TCMSP^TM^). Afterward, three important *in silico* ADME indexes including OB (oral bioavailability), Caco-2 (cell permeability), and DL (drug-likeness) were employed to screen the candidate active ingredients (Cao et al., 2015). The threshold values of these three indexes were set as follows: OB ≥ 30%, Coca-2 ≥-0.4, and DL ≥ 0.18, respectively (Liu et al., 2016). These ingredients, which met all of three criteria above, were selected as candidate molecules for further analysis. Moreover, some ingredients were also supplemented manually by a wide-scale data-mining way.

#### Targets Fishing and Network Construction

A comprehensive approach of information integration, chemometric method and text-mining was introduced to discover the targets of XSHG. Firstly, the most likely biological targets were retrieved from PharmMapper sever (Yue et al., 2017a), STITCH^3^, and SEA^4^ (Keiser et al., 2007; Liu et al., 2010; Kuhn et al., 2012). Afterward, the candidate bioactive ingredients were imported to Google Scholar, DrugBank^5^, TTD^6^, and Herbal Ingredients Targets database (HIT) (Yue et al., 2017a) to discover the relevant targets supported by literature. Besides, to better understand the mechanism of XHSG for the treatment of anemia, all obtained targets were also sent to TTD, PharmGKB^7^, and Comparative Toxicogenomics Database (CTD^8^) (Ye et al., 2011; Zhu et al., 2012). And targets which were implicated in the clinical manifestations of anemia were retained, while others were eliminated. Finally, the signaling pathways related to anemia were extracted from KEGG database, and compound-target-pathway (C-T-P) network was generated by using Cytoscape 3.0 software.

### Experimental Validation

#### Analysis of Key Targets

Based on the results of metabolomics and network pharmacology, we focused on their shared pathways to discover the potential mechanisms. The metabolites of these pathways could be found, then metabolite-protein interactions from HMDB, protein–protein interactions from KEGG, and the relevant interaction genes in STRING could be further induced. As a result, the network containing relationships among metabolites, enzymes, pathway-related proteins and relevant interaction genes was established. By analyzing this network, the key targets in this interaction network were selected for experimental validation. Furthermore, other significant targets closely related to anemia were also added based on the previous reports. Collectively, these targets obtained above were validated by ELISA and Western blot analysis.

#### ELISA Analysis

Plasma levels of TNF-α, IL6, F2, and EPOR in different groups were measured by commercial ELISA kits (R&D Systems, United States) according to the protocols provided from the manufacture. Absorbance was detected at 450 nm by the EnSpire^TM^ microplate reader (PerkingElmer, United States).

#### Western Blot

To extract the protein of liver, the liver samples were homogenized with whole lysis buffer (1 mmol/L phenylmethylsulfonyl fluoride, 10 mmol/L Tris-HCl, 30 mmol/L sodium pyrophosphate, 50 mmol/L sodium fluoride, 250 mmol/L sodium chloride, 0.5% Triton X-100, 10% glycerol, 1 × proteinase inhibitor mixture, 2 mmol/L iodoacetic acid, and 5 mmol/L ZnCl_2_). Western Blot assays of ACSS1, COASY, CBS, AHCY, SPHK1, S1PR1, and HIF-1α (Proteintech, United States) were carried out by standard protocols based on the manufacturer’s instructions.

### Statistics

Statistical analysis was performed by using SPSS 19.0 software, all data were expressed as mean ± standard deviation. The statistical results were conducted with two-tailed unpaired Student’s *t*-test, and value of *P* ≤ 0.05 was regarded significant difference.

## Results

### Peripheral Blood Routine Analysis

The peripheral blood routine analysis was firstly evaluated to test whether the anemia model was constructed successfully. As shown in **Table 1**, compared with control group, HGB, RBC, and LY% in model group were obviously reduced (*P* < 0.01), while PLT, WBC, HCT, MO%, and GR% in model group were significantly increased (*P* < 0.01). The results indicated that anemia model was successfully induced. The levels of PLT, WBC, HCT, MO, and GR in XSHG were significantly reduced compared with model group (*P* < 0.05), whereas the decreased HGB, RBC, and LY% in model group were obviously elevated after oral administration of XSHG (*P* < 0.01). The MS data were successfully submitted to the online MassIVE datasets^9^, and the accession number was MSV000081992.

**Table 1:** Effects of XSHG after 12 days administration on peripheral blood parameters of anemia rats *(-X ± SD, n = 10).*

Parameter	WBC (10^9^/L)	RBC (10^12^/L)	HGB (g/L)	HCT (%)	PLT (10^9^/L)	LY (%)	MO (%)	NE (%)
Control	18.22 ± 2.04	6.22 ± 0.49	143.39 ± 4.72	33.56 ± 2.78	935.33 ± 45.85	86.54 ± 1.66	6.87 ± 0.96	2.28 ± 0.38
Model	24.51 ± 0.98^∗∗^	2.99 ± 0.20^∗∗^	83.32 ± 7.22^∗∗^	21.35 ± 3.74^∗∗^	1302.33 ± 120.32^∗∗^	77.78 ± 3.25^∗∗^	11.40 ± 2.39^∗∗^	1.20 ± 0.29^∗∗^
XSHG	19.15 ± 3.18^##^	4.03 ± 0.21^##^	106.67 ± 4.89^##^	27.23 ± 1.52^##^	1139.22 ± 110.37^#^	83.63 ± 2.59^##^	8.04 ± 1.06^##^	1.57 ± 0.17^#^

*^∗^P < 0.05, ^∗∗^P < 0.01 vs. control group; ^#^P < 0.05, ^##^P < 0.01 vs. model group.*

*WBC, white blood cell count; RBC, red blood cell count; HGB, hemoglobin; HCT, haematocrit; PLT, platelet count; LY, lymphocyte ratio; MO, monocyte ratio; NE, neutrophils ratio.*

### Results of Plasma Metabolomics

#### Metabolomics Profiling of Plasma Samples

**FIGURE 2:**
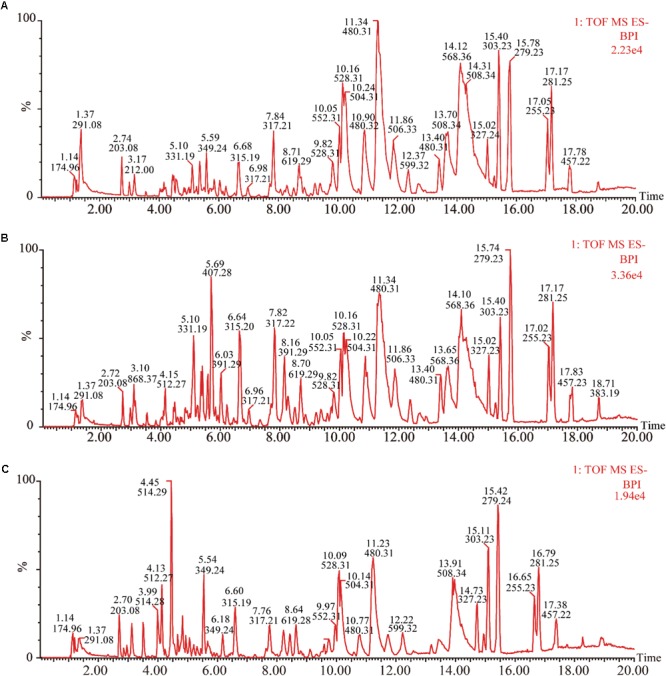
Representative base peak intensity chromatograms of plasma samples of different groups derived from UHPLC-QTOF/MS. **(A)** Control group, **(B)** model group, and **(C)** XSHG group.

Under the optimized chromatography conditions, the typical based peak intensity (BPI) chromatograms of plasma samples were analyzed in both positive and negative modes (**Figure 2**). And the endogenous markers obtained excellent separation within 20 min. Among different groups, the subtle changes from complex MS data could be discovered through multivariate data analysis methods, including PCA, PLS-DA, and OPLS-DA.

#### Multivariate Data Analysis

**FIGURE 3:**
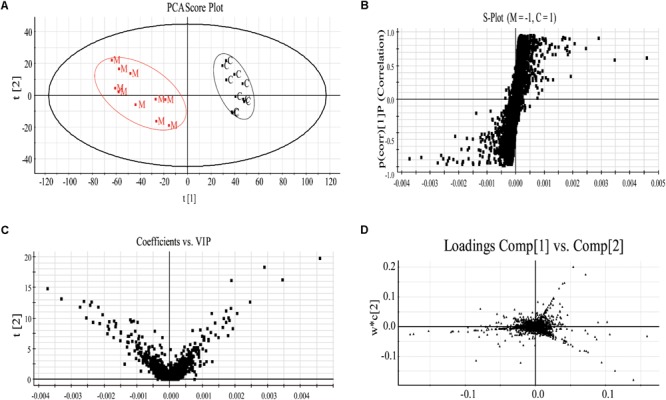
The plasma PCA score plot **(A)**; S-plot of OPLS-DA **(B)**; and VIP-plot of OPLS-DA **(C)** between control and model groups and loading plot of PLS-DA **(D)** of control, model and XSHG groups in negative mode (*n* = 10).

Specifically, to observe the subtle changes, raw data from UHPLC-MS were imported into EZinfo 2.0 software. Then an obvious separation in the PCA score plots between the model and control groups was clearly observed, indicating that biochemical perturbation occurred in the anemia group (**Figure 3A**). Then the S-plot of PLS-DA (**Figure 3B**) and VIP-value plot (**Figure 3C**) were drawn to unveil the metabolic biomarkers of anemia. Generally, the variables of VIP plots exhibiting values larger than 1 could be considered as the potential biomarkers, which were responsible for the differences between model and control groups (Wang et al., 2016a). The potential endogenous markers could also be discovered from the loading plot of the PLS-DA (**Figure 3D**). R^2^Y of the PLS-DA model in positive and negative modes was 0.996 and 0.978, respectively; *Q*^2^ was 0.938 and 0.855 accordingly, which showed that this PLS-DA model was excellent for prediction and fitness.

Moreover, the relative distances from treatment groups (model and XSHG groups) to control group in the PLS-DA score plot were applied for quantitation (Supplementary Figure S1). And the mean metabolic patterns of normal rats were defined as the jumping-off point of other groups (Duan et al., 2016). In comparison with model group, the relative distance of XSHG group decreased obviously in both positive and negative modes, demonstrating that XSHG could adjust the anemia to the normal (**Table 2**).

**Table 2:** Relative distance between drug groups (model and XSHG) and control group from the PLS-DA score plot of the plasma samples *(± SD, n = 10).*

Sample	ESI mode	Control		Model	XSHG
		*x*-axis	*y*-axis		
Plasma	+	42.81	–48.35	99.58 ± 7.39	29.73 ± 7.62^##^
	–	33.28	26.83	92.35 ± 11.88	23.56 ± 4.32^##^

*^#^P < 0.05, ^##^P < 0.01 vs. model group.*

#### Identification of Endogenous Metabolites

The potential endogenous biomarkers could be identified based on MS/MS fragments, retention behavior and online databases query. Firstly, candidate biomarkers were extracted from S-plots and loading plots performed, following analysis of OPLS-DA from MarkerLynx (Wettersten and Weiss, 2013). The accurate molecular mass and *m/z* values of metabolites were then determined. Subsequently, we could predict these metabolites initially through querying some on-line databases (HMDB, KEGG, and METLIN). And the potential markers were analyzed within an appropriate degree of measurement error (<10 ppm). Moreover, the MS/MS spectrum of different fragment ions were obtained in a targeted MS/MS mode. At last, these endogenous metabolites were identified by comparing with standard references and mass assignments in online databases (Xu et al., 2015).

Based on the protocols described above, a total of 18 endogenous biomarkers in plasma samples were tentatively identified (**Table 3**). LysoPC (20:4) and deoxycholic acid were identified by chemical standards, whereas other endogenous markers were identified tentatively by comparing accuracy mass information and corresponding assignments of product ions. To illustrate the identification process, one endogenous biomarker (Rt = 9.78, *m/z* = 543.6729) with VIP value of 8.03 was detailed as an example. Firstly, the exact mass of this biomarker ([M + H]^+^ at 544.0729) was obtained based on the mass spectrum in total ion chromatogram. Afterward, specific MS/MS fragments of this biomarker were determined by QTOF system. From the positive spectrum, main fragment ions of the candidate metabolite were observed at m/z 526.33, 484.29, 258.10, 184.07, 125.00, and 104.12, which could be the [M + H]^+^ of lost H_2_O, C_3_H_10_N, C_23_H_37_O_2_, C_26_H_44_O_3_, and C_24_H_42_O_4_P, respectively. Molecular formula C_28_H_50_NO_7_P was located as the potential marker by the use of measurement error of 5 ppm. Finally, this biomarker was identified as LysoPC (20:4) through querying HMDB and METLIN databases.

**Table 3:** The disturbed plasma endogenous metabolites between the control and anaemia rats as well as their identification results.

No	*T*_R_ (min)	m/z	VIP	Fold Change^∗^	Formula	Metabolites	ESI mode	Trend	Pathway	HMDB ID
Pm1	4.98	402.0862	7.47	3.84	C_12_H_23_N_2_O_9_PS	4-phosphopantothenoylcysteine	+	↑	a	01117
Pm2	5.07	332.2066	19.02	2.69	C_10_H_13_N_4_O_7_P	dIMP	+	↑	j	06555
Pm3	5.50	222.2621	5.25	3.02	C_7_H_14_N_2_O_4_S	L-cystathionine	+	↑	b, g, i	00099
Pm4	5.69	354.4813	4.00	1.65	C_20_H_34_O_5_	8-isoprostaglandin F2a	–	↑	–	05083
Pm5	6.56	316.4345	20.27	3.47	C_20_H_28_O_3_	15-deoxy-d-12,14-PGJ2	+	↑	–	05079
Pm6	6.89	318.4073	6.49	2.83	C_19_H_26_O_4_	Ubiquinone-2	+	↑	–	06709
Pm7	7.73	301.5108	6.90	1.65	C_18_H_39_NO_2_	Sphinganine	–	↑	c	00269
Pm8	8.23	379.4718	3.13	1.61	C_18_H_38_NO_5_P	Sphingosine 1-phosphate	+	↑	c	00277
Pm9	8.41	392.5720	11.62	3.72	C_24_H_40_O_4_	Deoxycholic acid	+	↑	–	00626
Pm10	9.08	316.4776	5.60	2.41	C_21_H_32_O_2_	Pregnenolone	+	↑	d	00253
Pm11	9.55	314.4186	5.88	3.67	C_20_H_26_O_3_	4-oxo-retinoic acid	+	↑	h	06285
Pm12	9.78	543.6729	8.03	1.38	C_28_H_50_NO_7_P	LysoPC (20:4)	–	↑	e	10395
Pm13	13.46	300.4351	4.21	3.54	C_20_H_28_O_2_	9-*cis*-retinoic acid	+	↑	–	02369
Pm14	14.12	278.3468	1.82	1.68	C_15_H_22_N_2_O_3_	Leucyl-phenylalanine	+	↑	–	13243
Pm15	14.28	302.4510	4.23	0.52	C_20_H_30_O_2_	Eicosapentaenoic acid	+	↓	f	01999
Pm16	14.68	328.4883	1.65	0.67	C_22_H_32_O_2_	Docosahexaenoic acid	+	↓	f	02183
Pm17	15.39	312.4458	5.16	0.55	C_21_H_28_O_2_	16-dehydroprogesterone	–	↓	–	00995
Pm18	18.16	384.4116	6.58	1.97	C_14_H_20_N_6_O_5_S	S-adenosylhomocysteine	+	↑	b	00939

*^∗^Fold change with a value > 1 indicates a relatively higher concentration present in the anemia model.*

*a Compared with control group: ↑, content increased; ↓, content decreased.*

*b(a) pantothenate and CoA biosynthesis, (b) cysteine and methionine metabolism, (c) sphingolipid metabolism, (d) steroid hormone biosynthesis, (e) glycerophospholipid metabolism, (f) biosynthesis of unsaturated fatty acids, (g) nitrogen metabolism, (h) retinol metabolism, (i) glycine, serine and threonine metabolism, (j) purine metabolism.*

Among the 18 endogenous metabolites, the levels of eicosapentaenoic acid, docosahexaenoic acid and 16-dehydroprogesterone in model group were up-regulated significantly (*P* < 0.05), whereas other biomarkers in model group were down-regulated obviously (*P* < 0.01). Furthermore, the relative intensities of 18 endogenous markers were determined for semi-quantitation, and it was discovered that the contents of these metabolites in XSHG group tended to the normal group (Supplementary Figure S2).

#### Metabolic Pathway Analysis

**FIGURE 4:**
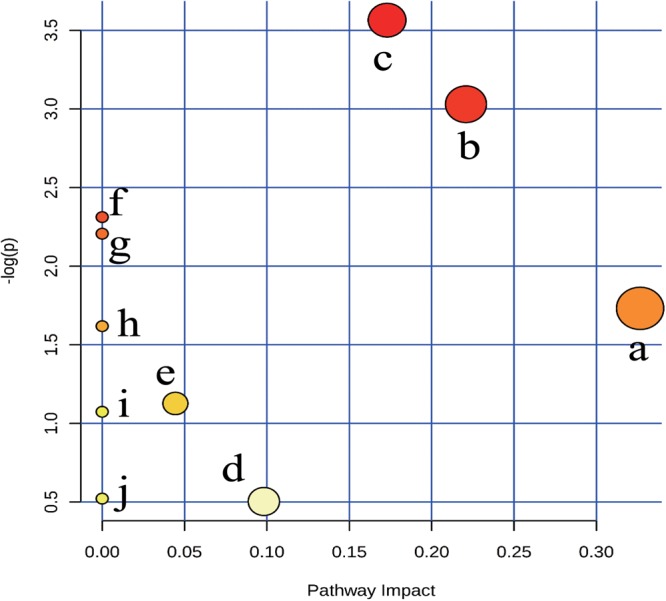
Summary of pathway analysis of plasma samples collected from anaemia rats. (a) pantothenate and CoA biosynthesis, (b) cysteine and methionine metabolism, (c) sphingolipid metabolism, (d) steroid hormone biosynthesis, (e) glycerophospholipid metabolism, (f) biosynthesis of unsaturated fatty acids, (g) nitrogen metabolism, (h) retinol metabolism, (i) glycine, serine and threonine metabolism;, (j) purine metabolism.

To explore the potential metabolic pathways of anemia, 18 endogenous biomarkers were then imported into MetaboAnalyst. Ten corresponding pathways were constructed, which could be highly related to the anemia (Supplementary Table S1). Among these metabolic pathways, pantothenate and CoA biosynthesis with impact-value 0.33, cysteine and methionine metabolism with impact-value 0.22, and sphingolipid metabolism with impact-value 0.17 were filtered out as significant pathways (**Figure 4**), for the reason that the threshold of the impact-value over 0.10 was usually considered as the most important metabolic pathways (Hoffmann et al., 2014).

Of the 18 potential metabolites identified from these pathways, several played important roles in the development of anemia. 4-Phosphopantothenoylcysteine was a key intermediate metabolite of coenzyme A (CoA), which could provide 90% of the body energy. Pantothenoylcysteine could be combined with triphosphate and adenosine to form CoA, could promote hematopoietic function through improving energy metabolism, activating the immune system, and increasing the synthesis of erythrocyte. In anemia patients, the activities of ATPase decreased obviously, which might result from the low levels of 4-phosphopantothenoylcysteine (Pietrocola et al., 2015). *S*-Adenosylhomocysteinase and L-Cystathionine were mainly involved in cysteine and methionine metabolism, and they could play key roles in cardiovascular disease and biosynthesis of folic acid (Pacana et al., 2015). The decreased contents suggested that cysteine and methionine metabolism was inhibited, besides, several decreased biomarkers like sphinganine, and sphingosine 1-phosphate could be found and applied to explain sphingolipid metabolism. Researches have demonstrated that sphingolipids played crucial roles in metabolic diseases by regulating cell proliferation, differentiation and death (Harvald et al., 2015). The contents of sphinganine and sphingosine 1-phosphate reduced in anemia, which could be caused by the injured membrane of erythrocyte and disordered peripheral blood parameters (Li et al., 2015).

#### Correlation Analysis Between Biochemical Indicators and Biomarkers

**FIGURE 5:**
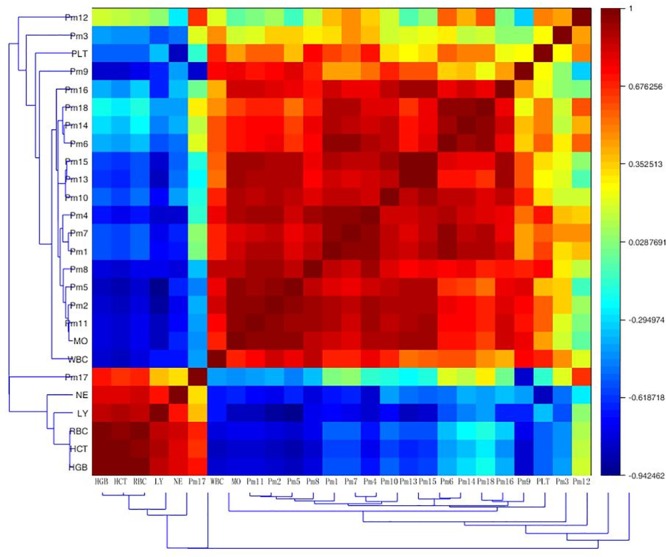
Correlation analysis between potential biomarkers and efficacy indicators of periphery blood parameters based on their Pearson correlation coefficient. Pm1, 4-phosphopantothenoylcysteine; Pm2, dIMP; Pm3, L-cystathionine; Pm4, 8-isoprostaglandin F2a; Pm5, 15-deoxy-d-12,14-PGJ2; Pm6, ubiquinone-2; Pm7, sphinganine; Pm8, sphingosine 1-phosphate; Pm9, deoxycholic acid; Pm10, pregnenolone; Pm11, 4-oxo-retinoic acid; Pm12, LysoPC (20:4); Pm13, 9-*cis*-retinoic acid; Pm14, Leucyl-phenylalanine; Pm15, Eicosapentaenoic acid; Pm16, docosahexaenoic acid; Pm17, 16-dehydroprogesterone; Pm18, *S*-adenosylhomocysteinase; WBC, white blood cell count; RBC, red blood cell count; HGB, hemoglobin; HCT, haematocrit; PLT, platelet count; LY, lymphocyte ratio; MO, monocyte ratio; NE, neutrophils ratio.

To test whether biochemical indicators have relations with biomarkers, the Pearson correlation matrix was applied for the correlation analysis. As was shown in **Figure 5**, there was a certain correlation between peripheral blood routine and endogenous metabolites in anemia rats. The markers 4-phosphopantothenoylcysteine, dIMP, 8-isoprostaglandin F2a, ubiquinone-2, sphinganine, sphingosine 1-phosphate, 4-oxo-retinoic acid and leucyl-phenylalanine were strong positive associated with WBC, PLT and MO (*r* > 0.5); 4-phosphopantothenoylcysteine, dIMP, 8-isoprostaglandin F2a, sphinganine, sphingosine 1-phosphate and 4-oxo-retinoic acid had strong negative association with RBC, HGB, HCT, LY, and NE (*r* < -0.5); additionally, 15-deoxy-d-12,14-PGJ2 had strong positive association with WBC and MO (*r* = 0.776, 0.996), as well as strong negative association with RBC, HGB, HCT, and LY (*r* = -0.737, -0.725, -0.509, -0.683). Deoxycholic acid was significantly positive associated with WBC and MO (*r* = 0.702, 0.780), and negative associated with RBC, HGB, and HCT (*r* = -0.803, -0.674, -0.643). The level of PLT was positive associated with LysoPC (20:4) (*r* = 0.503). Eicosapentaenoic acid showed strong positive association with WBC and LY (*r* = 0.560, 0.612). An obvious positive correlation was discovered between docosahexaenoic acid and MO (*r* = 0.745), and it was strong negative associated with LY (*r* = -0.535). These correlationships might be valuable for understanding the pathological process of anemia disease.

### Results of Network Pharmacology

#### Screening of XSHG Active Compounds

Although the TCM prescriptions or the single herbs usually contained thousands of constituents, only a few with satisfying pharmacokinetic and pharmacodynamics features contributed to its curative effects. Our previous work has shown that major bioactive compounds of XSHG were aromatic acids, phthalides, alkaloids, flavonoids, and gingerols (Pang et al., 2016a). In present study, three key ADME parameters, including OB, DL and Caco-2, were applied to mine the major active components in XSHG. Additionally, several active constituents which did not satisfy these three criteria above were also introduced due to relative high contents or excellent bioactivities.

First of all, a gross of 250, 102, 378, 132, 378, 296, and 560 candidate ingredients were obtained from Danggui, Yimucao, Chuanxiong, Taoren, Honghua, Jiangtan, and Zhigancao, respectively. Combined with the ADME profiles and literature confirmation, some bioactive ingredients could be screened from these compounds. The general filtering criteria were: OB ≥ 30%, DL ≥ 0.18, and Caco-2 ≥-0.4. Consequently, a total of 58 potential constituents were selected for further analysis (Supplementary Table S2). To evaluate whether these constituents could be detected in the rat plasma after oral administration of XSHG prescriptions, 43 ingredients were characterized by UHPLC-QTOF-MS, which suggested that these screening compounds were believable to be applied in the network pharmacology analysis. The numbers of bioactive constituents were 22 for ASD and LC, 15 for LA, 7 for PP, 5 for CT, 4 for RZR, and 5 for RGP, respectively. Among the 58 ingredients, most of them had desirable pharmacokinetic properties. For instance, ferulic acid (M1, OB = 39.56%, Caco-2 = 0.47, and DL = 0.18) has antibacterial, anti-inflammatory, antioxidant and radio-protective effects (Mancuso and Santangelo, 2014). It should be pointed out that stachydrine held low OB values (OB = 0.27), but the content of stachydrine was found to be about 10 mg/g in XSHG (Pang et al., 2016a), which possessed potent anti-inflammatory, antiplatelet aggregation and antioxidant activities (Li B.H. et al., 2013). Similarly, the phthalides, the marker constituents in ASD and LC, showed low DL values, but they exhibited remarkable pharmacological activities (Wei et al., 2013). Thus, these compounds were also selected for further analysis.

#### Target Proteins of XSHG

Subsequently, a comprehensive *in silico* method was intended to construct the protein targets of the 58 compounds. Integrated models contained PharmMapper Sever, STITCH and SEA. Other databases were also used, including Google Scholar, DrugBank, Therapeutic Targets Database, and Herbal Ingredients’ Targets database. As a result, 46 candidate targets related to anemia were tentatively fished out, generating 373 target-ligand interactions: 168 for ASD and LC, 100 for LA, 39 for PP, 22 for CT, 18 for RZR, and 26 for RGP. The average number of candidate targets for each constituent was 6.4. Among the 58 active compounds, 7 had high degrees, including ferulic acid (degree = 17), ligustilide (degree = 16), stachydrine (degree = 15), leonurine (degree = 14), amygdalin (degree = 13), hydroxysafflor yellow A (degree = 11), glycyrrhizic acid (degree = 10), which showed the poly-pharmacology and multi-target properties of this prescription. At the same time, our previous studies exhibited that these compounds had larger areas under the curve compared to others (Pang et al., 2017). Based on the above analysis, these compounds undoubtedly played crucial roles in the treatment of anemia. Target information of the 58 bioactive constituents was listed in **Table 4**.

**Table 4:** The protein targets information of 58 active constituents of XHSG.

Gene	Uniprot ID	Target	Degree	Gene	Uniprot ID	Target	Degree
TNF-α	P01375	Tumor necrosis factor	25	DCK	P27707	Deoxycytidine kinase	9
IL1β	P01584	Interleukin-1 beta	19	HMGCR	P04035	3-hydroxy-3-methylglutaryl-coenzyme A reductase	9
MAPK1	P28482	Mitogen-activated protein kinase 1	19	MMP9	P14780	Matrix metalloproteinase-9	9
MAPK10	P53779	Mitogen-activated protein kinase 10	18	SOD2	P04179	Superoxide dismutase (Mn)	9
IL6	P05231	Interleukin-6	17	TBXAS1	P24557	Thromboxane-A synthase	9
PTGS2	P35354	Prostaglandin G/H synthase 2	17	ESR2	Q92731	Estrogen receptor beta	8
MAPK14	Q16539	Mitogen-activated protein kinase 14	15	HIF1α	Q16665	Hypoxia-inducible factor 1-alpha	8
F2	P00734	Prothrombin	14	MMP2	P08253	Matrix metalloproteinase-2	8
NOS2	P35228	Nitric oxide synthase, inducible	12	PROCR	Q9UNN8	Endothelial protein C receptor	8
NOS3	P29474	Nitric oxide synthase, endothelial	12	TLR4	O00206	Toll-like receptor 4	8
EPOR	P19235	Erythropoietin receptor	11	CA3	P07451	Carbonic anhydrase 3	7
ESR1	P03372	Estrogen receptor	11	FGFR1	P11362	Fibroblast growth factor receptor 1	7
FGG	P02679	Fibrinogen gamma chain	11	LPCAT1	Q8NF37	Lysophosphatidylcholine acyltransferase 1	7
PCK1	P35558	Phosphoenolpyruvate carboxykinase	11	SOD1	P00441	Superoxide dismutase [Cu-Zn]	7
CA2	P00918	Carbonic anhydrase 2	10	VEGFA	P15692	Vascular endothelial growth factor A	7
CASP3	P42574	Caspase-3	10	CA1	P00915	Carbonic anhydrase 1	6
HMOX1	P09601	Heme oxygenase 1	10	FDPS	P14324	Farnesyl pyrophosphate synthase	6
IL2	P60568	Interleukin-2	10	KDR	P35968	Vascular endothelial growth factor receptor 2	6
GSTA1	P08263	Glutathione *S*-transferase A1	10	PAH	P00439	Phenylalanine-4-hydroxylase	6
PKLR	P30613	Pyruvate kinase PKLR	10	ALOX5	P09917	Arachidonate 5-lipoxygenase	5
AHCY	P23526	Adenosylhomocysteinase	9	G6PD	P11413	Glucose-6-phosphate 1-dehydrogenase	5
BCL2	P10415	Apoptosis regulator Bcl-2	9	HCK	P08631	Tyrosine-protein kinase HCK	5
CBS	P35520	Cystathionine beta-synthase	9	S1PR1	P21453	Sphingosine 1-phosphate receptor 1	5

Remarkably, multiple molecular targets were interacted with the bioactive constituents, such as TNF-α, IL-1β, MAPK1, MAPK10, IL-6, PTGS2, F2, HIF-1α, and EPOR. And the majority of 46 targets were directly related to anemia and vascular systems. For examples, TNF-α, IL-1β, and IL-6 could regulate the vascular systems to improve the vascular injury, and promote the hematopoietic function after postpartum hemorrhage (Janssens et al., 2015); MAPK1 and MAPK10, the mitogen-activated protein kinases, were involved in cell biological responses and immune defense by mediating multiple cellular processes (Mohanta et al., 2015); PTGS2 contributed to the development of thrombosis and atherosclerosis in response to the production of eicosanoids (Deeb et al., 2006). Moreover, stachydrine, leonurine, ferulic acid and *Z*-ligustilide might mediate the expressions of F2, HIF1α, and EPOR to improve blood circulation, thus exerting the effects of immune enhancement, erythrocyte synthesis and blood-vessel dilation (Bi et al., 2012). Senkyunolide I may interact with MAPK1, IL-1β, and TNF-α, which were also involved in anemia and vascular systems (Hu et al., 2016).

Except for β-sitosterol, nicotinic acid and vanillin, other bioactive constituents also interacted with protein targets relevant to pain, inflammation, microcirculation disorders, immune and nervous system diseases (Yue et al., 2017b). Five potential targets containing F2, FGG, MAPK14, TBXAS1, and ALOX5 were closely related to micro-circulation disorders (Yue et al., 2017b), which could explain why XSHG had the function of improving microcirculation. Besides, several targets such as CA2, HMOX1, GSTA1, and NOS2 were closely associated with anemia (Lin et al., 2013), which could exhibit significant influences on the therapeutic effects of XSHG.

#### C-T-P Network Analysis

**FIGURE 6:**
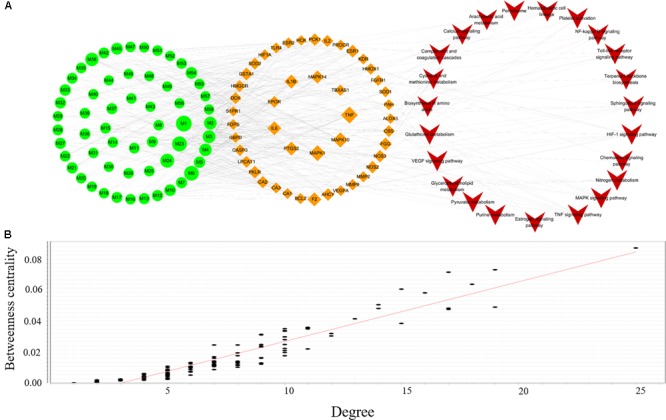
Compound-Target-Pathway network of active ingredients in XSHG for anemia disease **(A)**; the correlation plots of the degree and betweenness centrality for this network **(B)**. Fifty-eight active ingredients map 46 potential targets and 24 signaling pathways. Green nodes represent the ingredients, brown nodes represent the targets, and red nodes are pathways.

To clarify the underlying mechanism of XSHG, 24 anemia-relevant signaling pathways were obtained from KEGG. Among these pathways, seven were consistent with the results of metabolomics. Then we mapped all potential ingredients with their corresponding targets onto these predictable pathways. And a global view of C-T-P network was generated by Cytoscape 3.4.0, which consisted of 128 nodes (58 ingredients, 46 genes and 24 pathways) and 453 edges (**Figure 6A**). Besides, the relationships between betweenness centrality and degree in the C-T-P network (**Figure 6B**) demonstrated that the distribution of betweenness centrality and degree was strongly correlated and the most highly connected vertices had high centrality scores. Most pathways were interacted with a few targets, but about 20% candidate targets were involved in multi-pathways (≥5), reflecting the crucial roles in treating anemia. For example, NOS3, which was closely associated with immune enhancement, vasodilation and neuroprotective effects (Shu et al., 2015), was influenced by six constituents through six pathways.

Moreover, we discovered that four biological pathways, i.e., HIF-1α, TNF, MAPK, and sphingolipid signaling pathways were remarkably enriched for the mined targets, implying the significant roles of these potential pathways. Among them, HIF-1α signaling pathway had the highest degree and the crucial targets in this pathway such as HMOX1, HIF-1α, F2, and IL6 were highly related to the hematopoietic function (Haase, 2013). Also, it could be observed that most compounds of XHSG were involved in these signaling pathways, providing a scientific basis for prevention and treatment of anemia. For example, 33 compounds such as *Z*-ligustilide, ferulic acid, and stachydrine were referred to mediating key targets in HIF-1α signaling pathway.

Furthermore, five biological pathways containing VEGF, Estrogen, Toll-like receptor, NF-kB, and hematopoietic cell lineage signaling pathways were the second major pathways able to exhibit anti-oxidative, anti-inflammatory, immune responses, and neuroprotective effects (DiDonato et al., 2012; Cabas et al., 2013; Maglione et al., 2015). It should be noted that different types of active ingredients could interact with various pathways. For alkaloids, VEGF, Estrogen and Toll-like receptor, signaling pathways exhibited higher correlations with their corresponding target proteins, whereas phthalides were mainly involved in HIF-1α and hematopoietic cell lineage pathways. These results indicated that various constituents of XHSG might act on different biological pathways.

### Results of Experimental Validation

#### Selection of Crucial Targets

**FIGURE 7:**
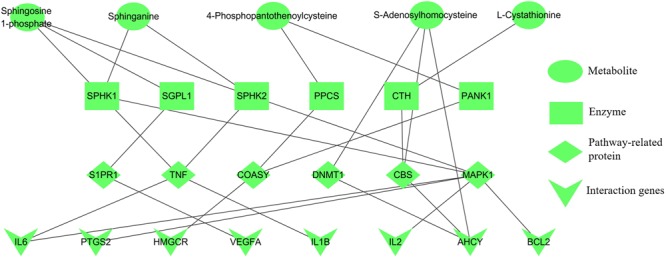
Five candidate metabolites for anemia associated with relevant protein targets. Metabolites (green), enzymes (brown), pathway-related proteins (purple), and interaction genes (red).

Integrating the results of metabolomics and network pharmacology, the metabolite-protein interaction network, containing five metabolites, six enzymes, six pathway-related proteins and eight interaction genes, was constructed (**Figure 7**). Then the crucial targets of shared pathways were firstly selected from the constructed network: coenzyme A biosynthesis (ACSS1 and COASY), cysteine and methionine metabolism (CBS and AHCY), and sphingolipid signaling pathway (SPHK1 and S1PR1). Moreover, other key targets, which were closely associated with the erythrocyte function (EPOR and HIF-1α) and blood microcirculation (TNF-α, IL6, and F2), were also applied for the experiment validation. As shown in Supplementary Figure S3, the correlation plots of the degree and betweenness centrality indicated that targets AHCY, CBS, EPOR, IL6, and TNF had the relatively high connected vertices compared with the average values, suggesting these targets had the significant roles.

#### ELISA Analysis

**FIGURE 8:**
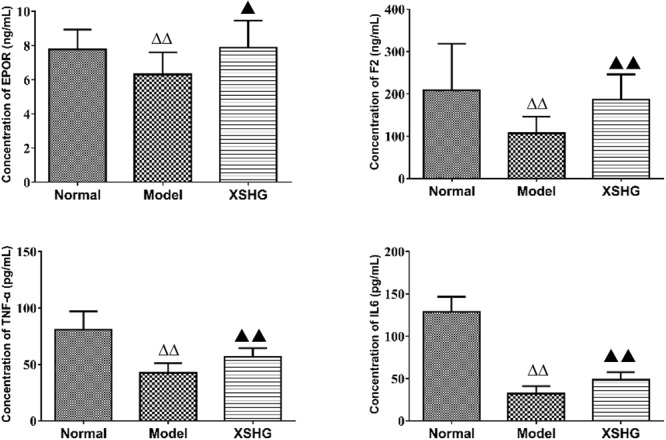
Expression of EPOR, F2, TNF-α, and IL-6 protein levels in rat plasma using ELISA analysis. Data were presented as the mean ± SD (*n* = 6), ^Δ^*P* < 0.05, ^ΔΔ^*P* < 0.01 vs. normal group; ▲*P* < 0.05, ▲*P* < 0.01 vs. model group.

As shown in **Figure 8**, the levels of TNF-α, IL6, F2, and EPOR were significantly reduced (*P* < 0.01) in the model, presenting the inhibition of these cytokines. After oral administration of XSHG, the decreased levels of TNF-α, IL6, F2, and EPOR were obviously increased (*P* < 0.05). TNF-α could inhibit erythropoiesis through inducing dendritic cell proliferation (Mizrahi et al., 2013), and the plasma TNF-α level might show a compensatory decrease in anemia rats. IL6 had the function of hematopoiesis, immune response and inflammation (Janssens et al., 2015). XSHG could raise the level of IL6 and promote the hematopoietic and immune function. Moreover, XHSG could improve the erythrocyte immune functions and wound healing by enhancing the expression of EPOR and F2 in anemia.

#### Western Blot Analysis

**FIGURE 9:**
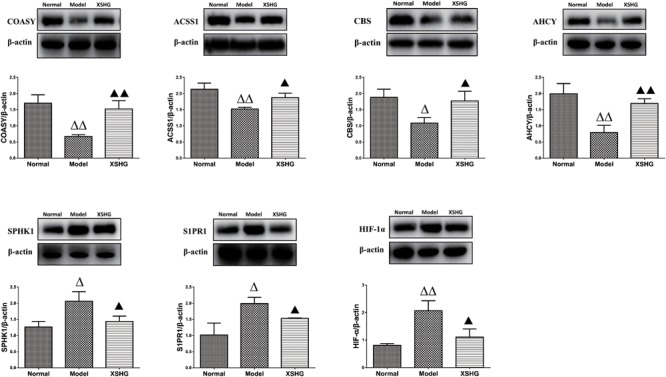
Western blot analysis of ACSS1, COASY, AHCY, CBS, S1PR1, SPHK1, and HIF-1α protein levels in rat liver. Data were presented as the mean ± SD (*n* = 6), ^Δ^*P* < 0.05, ^ΔΔ^*P* < 0.01 compared to normal group; while ▲*P* < 0.05, ▲▲*P* < 0.01 compared to the model group.

As we all know, the liver serves as the main hematopoietic organ in mammals, so certain enzymes undoubtedly play significant roles in hematopoietic function. ACSS1 and COASY levels in liver were firstly investigated to explore the effects of XSHG on energy metabolism, and Western blot results were exhibited in **Figure 9**. As expected, anemia rats showed obvious inhibition of ACSS1 and COASY. However, XSHG enhanced the expression of these targets, indicating that it could improve energy metabolism through activating coenzyme A biosynthesis (Tomita et al., 2013). We also explored whether XSHG could mediate the cysteine and methionine pathway in this model. Clearly, the reduced levels of AHCY and CBS were observed in anemia. After oral administration of XSHG, the levels of these two protein targets were markedly increased. CBS and AHCY mainly participated in DNA methylation, which could promote the biosynthesis of erythrocyte (Amabile et al., 2015). Consequently, XSHG might up-regulate cysteine and methionine pathway to improve erythrocyte function.

Furthermore, crucial targets of sphingolipid signaling pathway were investigated for the liver-protective effects of XSHG, and the expression of SPHK1 and S1PR1 was evaluated. The results indicated that XSHG could significantly reduce the expression of disturbed SPHK1 and S1PR1 to improve signal transduction (Harvald et al., 2015). Besides, to evaluate whether XSHG had the anti-anoxia effects, the expression of HIF-1α was determined in anemia rats. As expected, XSHG indeed down-regulated the protein level of HIF-1α, which could explain why HIF-1α pathway was blocked in XSHG group.

## Discussion

According to the above results, we assumed that bioactive compounds of XSHG might simultaneously interact with multiple pathways like coenzyme A biosynthesis, sphingolipids and HIF-1α biological pathways, thereby exhibiting synergistic effects in anemia disease. In fact, lots of researches have indicated the significant roles of HIF-1α, coenzyme A biosynthesis and sphingolipids biological pathways in treating anemia and related diseases.

As is well known, in anemia patients, the balance of internal environment is weakened, thus the energy and substance metabolism is disturbed, and it will lead to the aggravation of anemia disease. Coenzyme A, a significant coenzyme participated in the biosynthesis of fatty acid and pyruvate, had the effects of immune enhancement and tissue repair, thus exerting a vital role in the energy metabolism of body (Tomita et al., 2013). Several coenzyme A biosynthesis target genes, including ACSS1, COASY, and PKLR, were particularly important for improving the disordered energy metabolism. By activating coenzyme A biosynthesis, disturbed energy metabolism could be improved, thus to enhance the synthesis of erythrocyte. Recent studies have demonstrated that Ge-Gen-Qin-Lian Decoction could regulate energy metabolism to treat metabolic diseases through activation of coenzyme A biosynthesis (Zhang et al., 2016), as confirmed by our results.

Moreover, after hemorrhage, the numbers of erythrocyte decreased significantly. Correspondingly, the levels of several cytokines such as EPOR, F2, and IL6 were reduced, leading to the occurrence of hypoxia. HIF-1α, a mainly mediator of maintaining the oxygen balance, could mediate the generation and survival of circulating erythrocyte (Rankin et al., 2012). Due to the increased expression of EPOR, the hypoxia could be improved, which could explain why the protein level of HIF-1α in XSHG group was down-regulated. By blocking HIF-1α signaling pathway, XSHG could maintain the balance of active oxygen metabolism, thereby promoting cell differentiation and enhancing the generation of EPOR. Some researchers have revealed that Herba Leonuri obviously increased the numbers of erythrocyte, attenuated the levels of EPOR and IL6, inhibited HIF-1α expression, and consequently improved the anemia, which supported its crucial role in the improvement of anemia (Li X. et al., 2013).

Sphingolipids, the critical composition of cell membrane, could regulate the cellular growth, differentiation and aging (Testai et al., 2014). They included sphingomyelin, cerebroside, and ganglioside, among which, sphingomyelin might play significant roles in the acute leukemia disease (Li et al., 2015). Some studies have indicated that sphingolipids were involved in inflammation, apoptosis, cellular immunity response (Testai et al., 2014). During the process of anemia, the membrane of erythrocyte was injured, resulting in erythrocyte apoptosis. So far, more and more evidence has shown that organisms could initiate erythrocyte apoptosis to maintain homeostasis of anemia patients (Knapp et al., 2012; Egler and Lang, 2017). Our research group have confirmed that herb pair Danggui-Chuanxiong could suppress erythrocyte apoptosis by inhibiting sphingolipids dependent signaling molecules (Li W.X. et al., 2014). Thus, the levels of sphingolipids decreased in anemia, which was consistent with the injured membrane of erythrocyte and disordered peripheral blood indicators.

**FIGURE 10:**
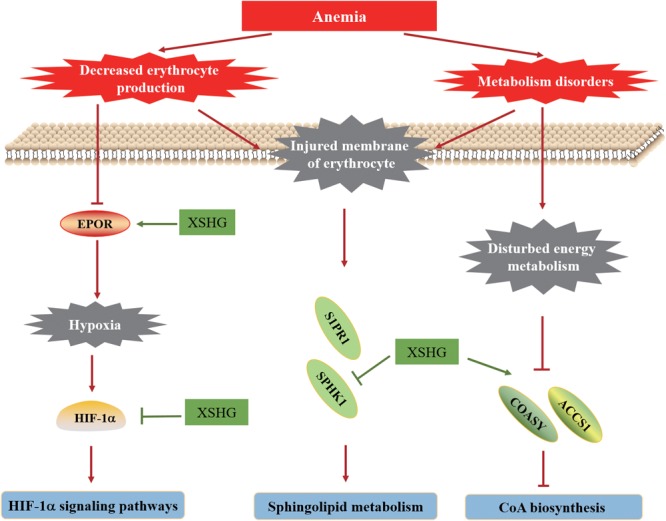
Hypothetical molecular mechanisms of XSHG for treating postpartum anaemia. Lines with arrow-heads represent activation, and lines with bars at the end denote inhibition.

In this work, an integrative approach of metabolomics and network pharmacology was performed to investigate the biological mechanisms of XSHG for treating anemia. Since the main ingredients of XSHG were aromatic acids, phthalides and alkaloids, we assumed that the treatments of XSHG might be ascribed to the integrate effects of multiple compounds rather than single constituent. The main action mechanisms of its therapeutic effects on anemia were shown in **Figure 10**. Collectively, the action mechanisms for treating anemia of XSHG were attributed to the activation of coenzyme A biosynthesis, inhibition of sphingolipid metabolism and suppression of HIF-1α pathway. And XSHG could play critical roles in anemia via increasing the expression of erythrocyte, improving the substance metabolism of liver, and promoting blood circulation. Further studies should evaluate the efficiency of the main active constituents such as stachydrine, ligustilide, and ferulic acid on other pathways and their interactions. This investigation was valuable for performing a systematical investigation of herb medicines, as well as for efficiently predicting the therapeutic targets of TCM formulae.

## Author Contributions

Y-PT and J-AD proposed the idea and designed this experiment. H-QP, Y-YC, Y-JT, and Y-JC performed the experiments. H-QP and S-JY designed the study. H-QP, X-QS, G-SZ, and AK participated in data analysis. S-JY, S-LH, Y-JS, JS, and Z-ST contributed to writing, revision and proof-reading this manuscript. All authors read and approved the final manuscript.

## Conflict of Interest Statement

The authors declare that the research was conducted in the absence of any commercial or financial relationships that could be construed as a potential conflict of interest. The reviewer P-MA and handling Editor declared their shared affiliation.

## Footnotes

1 http://www.stats.gov.cn/

2 http://lsp.nwu.edu.cn/tcmsp.php

3 http://stitch.embl.de/

4 http://sea.bkslab.org/

5 http://www.drugbank.ca/

6 http://bidd.nus.edu.sg/group/ttd/

7 http://www.pharmgkb.org

8 http://ctdbase.org/

9 https://massive.ucsd.edu/ProteoSAFe/static/massive.jsp
