# Supplementary material for: Integrated Metabolomics and Network Pharmacology Approach to Explain Possible Action Mechanisms of Xin-Sheng-Hua Granule for Treating Anemia
Source: Front Pharmacol. 2018 Mar 2;9:165. doi: 10.3389/fphar.2018.00165 (PMC5840524; doi:10.3389/fphar.2018.00165)
Supplement: Supplementary file 1 [file Presentation_1.pdf]

Integrated metabolomics and network pharmacology approach to explain possible action mechanisms of Xin-Sheng-Hua Granule for treating anaemia

***Han-Qing Pang*[1,2], *Shi-Jun Yue*[2], *Yu-Ping Tang*[1,2*], *Yan-Yan Chen*[1], *Ya-Jie Tan*[2], *Yu-Jie Cao*[2], *Xu-Qin Shi*[2], *Gui-Sheng Zhou*[2], *An Kang*[2], *Sheng-Liang Huang*[3], *Ya-Jun Shi*[1], *Jing Sun*[1], *Zhi-Shu Tang*[1], *Jin-Ao Duan*[2]**

[1]College of Pharmacy and Shaanxi Collaborative Innovation Center of Chinese Medicinal Resources Industrialization, Shaanxi University of Chinese Medicine, Xi'an 712046, Shaanxi Province, China, [2]Jiangsu Collaborative Innovation Center of Chinese Medicinal Resources Industrialization, and Jiangsu Key Laboratory for High Technology Research of TCM Formulae, and National and Local Collaborative Engineering Center of Chinese Medicinal Resources Industrialization and Formulae Innovative Medicine, Nanjing University of Chinese Medicine, Nanjing 210023, Jiangsu Province, China, [3]Jiangsu Revolence Pharmaceutical Co., Ltd., Huaian 223200, Jiangsu Province, China

** **Correspondence:***

Yu-Ping Tang

2051001@sntcm.edu.cn (Y.-P. Tang).

Supporting information:

TABLE S1

The metabolomics pathways of the plasma samples from blood deficiency rats.

TABLE S2

Active ingredients and ADME parameters of formulae XSHG. (ASD: Dangui; CR: Chuanxiong; LH: Yimucao; PS: Taoren; CF: Honghua; RZR: Jiangtan; GRP: Zhigancao).

FIGURE S1 PLS-DA score plot of the control, model, and XSHG groups on day 12 both in positive (A) and negative (B) modes.

FIGURE S2 The relative intensities of 18 endogenous biomarkers in control, model and XSHG groups. ($\overline{X} \pm SD$, n = 10) $^{\#}P < 0.05$, $^{\#\#}P < 0.01$ compared to control group; while $^{*}P < 0.05$, $^{**}P < 0.01$ compared to model group.

FIGURE S3 The correlation plots of the degree and betweenness centrality for targets AHCY, CBS, S1PR1, EPOR, IL6 and TNF.

TABLE S1

The metabolomics pathways of the plasma samples from blood deficiency rats.

**Pathway Name**	**Total Compounds**	**Hits**	**Raw p**	**Holm p**	**Impact**
Pantothenate and CoA biosynthesis	15	1	0.1770	1.0	0.3265
Cysteine and methionine metabolism	28	2	0.0483	1.0	0.2208
Sphingolipid metabolism	21	2	0.0283	1.0	0.1729
Steroid hormone biosynthesis	70	1	0.6045	1.0	0.0985
Glycerophospholipid metabolism	30	1	0.3241	1.0	0.0444
Biosynthesis of unsaturated fatty acids	42	2	0.0991	1.0	0.0000
Nitrogen metabolism	9	1	0.1101	1.0	0.0000
Retinol metabolism	17	1	0.1982	1.0	0.0000
Glycine, serine and threonine metabolism	32	1	0.3418	1.0	0.0000
Purine metabolism	68	1	0.5936	1.0	0.0000

TABLE S2

Active ingredients and ADME parameters of formula XSHG. (ASD: Dangui; LC: Chuanxiong; LA: Yimucao; PP: Taoren; CT: Honghua; RZR: Jiangtan; RGP: Zhigancao).

No	Name	Structure	OB (%)	Caco-2	DL	Herb
1	Ferulic acid*		39.56	0.47	0.18	ASD, LC
2	Caffeic acid*		54.97	0.27	0.19	ASD, LC
3	Chlorogenic acid*		11.93	-1.03	0.33	ASD, LC
4	Coniferyl ferulate*		4.54	0.71	0.39	ASD, LC
5	*p*-Coumaric acid*		43.29	0.46	0.04	ASD, LC

6	*Z*-ligustilide*	53.72	1.30	0.07	ASD, LC
7	*Z*-butylidenephthalide*	42.44	1.32	0.07	ASD, LC
8	Senkyunolide B*	43.18	1.01	0.08	ASD, LC
9	Senkyunolide A*	26.56	1.30	0.07	ASD, LC
10	Senkyunolide I*	46.80	0.87	0.08	ASD, LC

11	Senkyunolide H	41.04	0.91	0.09	ASD, LC
12	Tetramethylpyrazine	20.01	1.19	0.03	LC
13	Levistilide A*	16.56	0.83	0.05	LC
14	Neocnidilide	83.83	1.23	0.07	ASD, LC
15	Nicotinic acid	47.65	0.34	0.02	ASD, LC
16	Nodakenin	57.12	-0.79	0.69	ASD, LC

17	Vanillin	52.00	0.68	0.03	ASD, LC
18	Wallichilide*	42.31	0.82	0.71	LC
19	Perlolyrine	65.95	0.88	0.27	LC
20	Senkyunone*	47.66	1.15	0.24	LC
21	Kaempferol*	41.88	0.26	0.24	ASD, LC

22	6-Hydroxykaempferol*	62.13	0.16	0.27	ASD, LC
23	Stachydrine*	0.27	0.74	0.03	LA
24	Leonurine*	19.12	0.23	0.20	LA
25	Leonurinine*	2.60	-1.72	0.33	LA
26	Trigonelline*	60.07	0.58	0.03	LA
27	Syringic acid	47.78	0.50	0.06	LA

28	Ajugol*	16.87	-1.03	0.32	LA
29	Hispanolone*	18.89	0.90	0.26	LA
30	Prehispanolone	16.56	0.83	0.21	LA
31	Preleoheterin	85.97	0.46	0.33	LA
32	Isorhamnetin*	49.60	0.31	0.31	LA

33	Quercetin*	46.43	0.05	0.28	LA
34	Quercetin 3-rutinoside*	13.66	0.79	0.44	LA
35	Galeopsin*	61.02	0.42	0.38	LA
36	Wogonin*	30.68	0.79	0.23	LA

37	Daidzein	19.44	0.59	0.19	LA
38	Amygdalin*	4.42	-1.91	0.61	PP
39	Prunasin*	12.61	-0.79	0.18	PP
40	Benzoic Acid*	30.15	0.39	0.03	PP
41	Cianidanol*	54.83	-0.03	0.24	PP

42	*β*-Sitosterol*	33.94	-0.44	0.70	PP
43	Campesterin*	37.58	1.34	0.71	PP
44	Hederagenin	36.91	1.32	0.75	PP

45	Hydroxysafflor yellow A*	4.77	-2.77	0.68	CT
46	Anhydrosafflor yellow B*	2.34	-2.08	0.52	CT
47	Safflor yellow A*	22.75	-2.52	0.75	CT

48	Safflor yellow B	19.03	-2.08	0.69	CT
49	Carthamone*	5.93	-1.81	0.63	CT
50	6-Gingerol*	35.64	0.54	0.16	RZR
51	8-Gingerol	9.99	-1.91	0.09	RZR

52	10-Gingerol	19.14	0.48	0.28	RZR
53	Zingerone	25.23	0.87	0.05	RZR
54	Glycyrrhizic acid*	19.62	-2.66	0.11	RGP
55	Glycyrrhetinic acid*	22.05	0.10	0.74	RGP
56	Liquiritin*	13.01	-1.08	0.71	RGP

57	Isoliquiritin*	8.61	-1.36	0.60	RGP
58	Liquiritigenin	32.76	0.51	0.18	RGP

* The bioactive constituents were detected in rat plasma after oral administration of XSHG prescriptions by UHPLC-QTOF-MS.

OB: oral bioavailability, Caco-2: Caco-2 permeability, DL: druglikeness, ASD: the active compounds from Danggui (the radix of *A. sinensis*), LA: the active compounds from Yimucao (the herbs of *L. artemisia*), LC: the active compounds from Chuanxiong (the radix of *L. chuanxiong*), PP: the active compounds from Taoren (the seeds of *P. persica*), CT: the active compounds from Honghua (the florets of *C. tinctorius*), RZR: the active compounds from Jiangtan (the roasted rhizome of *Zingiber officinale* Rosc.), RGP: the active compounds from Zhigancao (Radix *Glycyrrhizae preparata*).

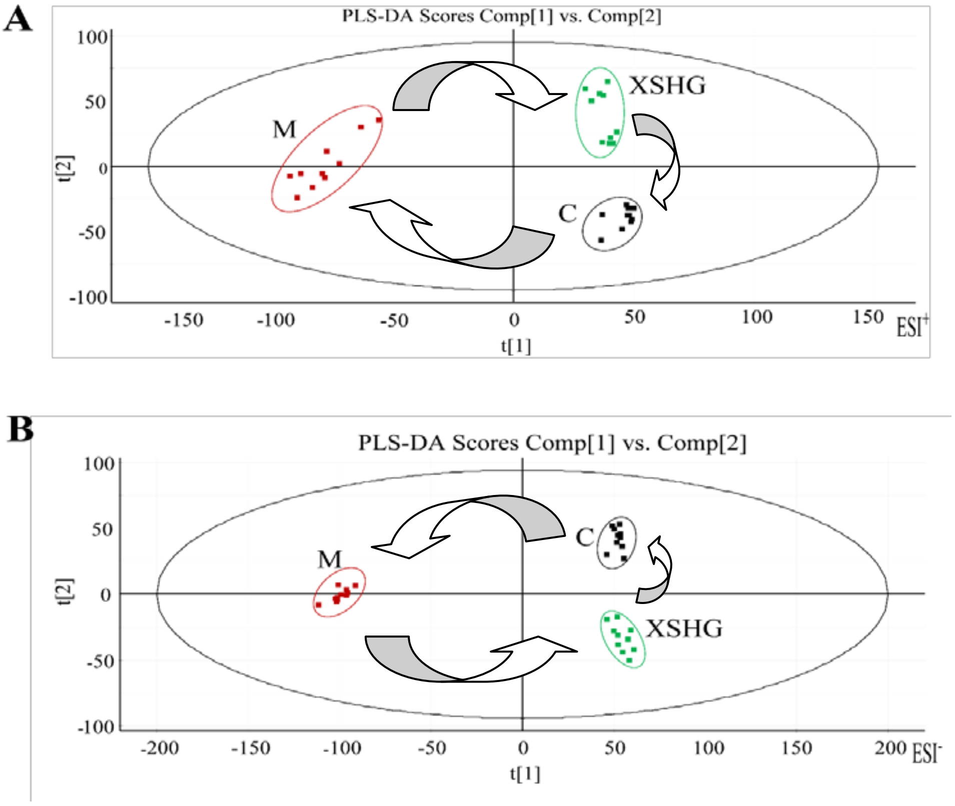

FIGURE S1 PLS-DA score plot of the control, model, and XSHG groups on day 12 both in positive (A) and negative (B) modes.

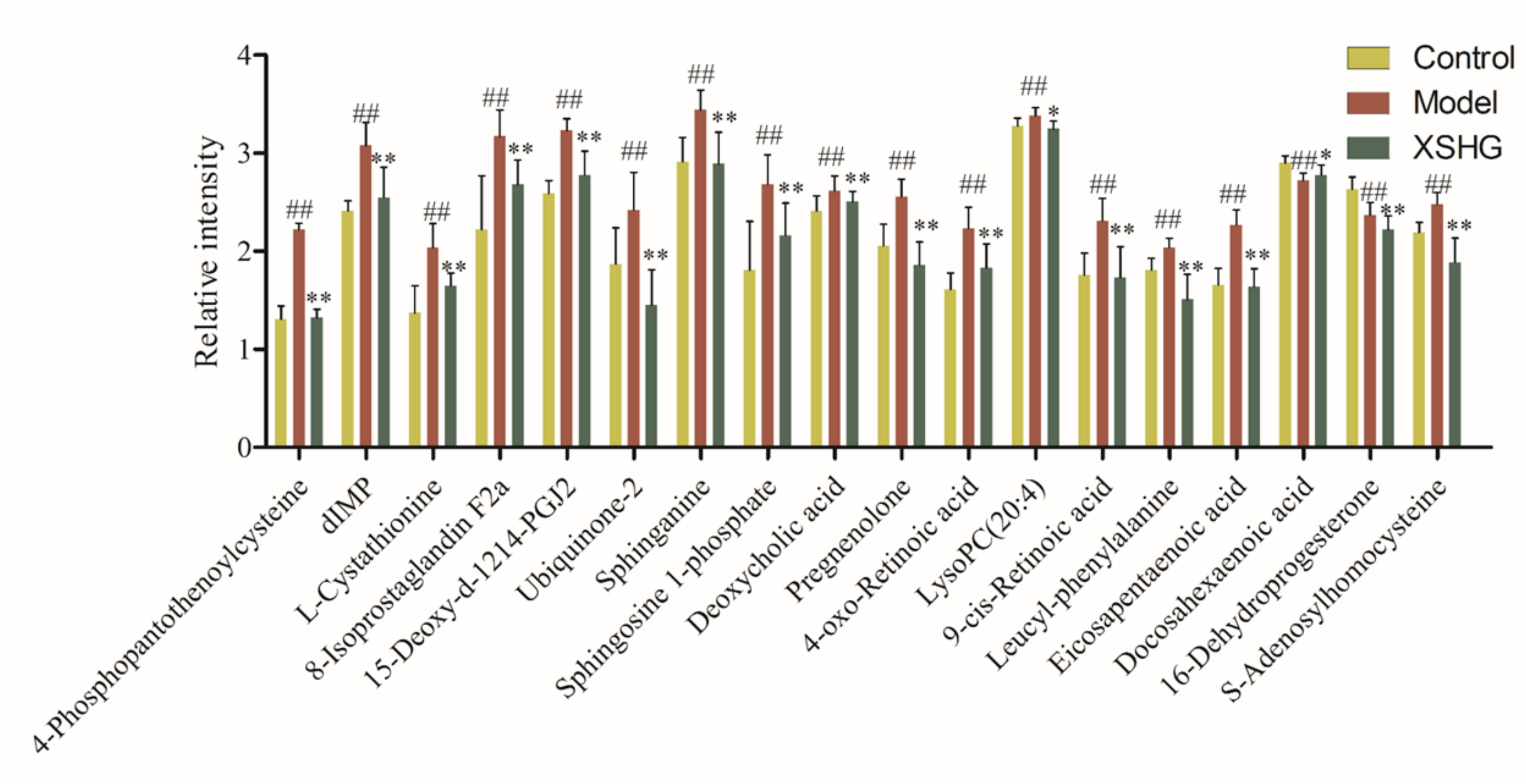

FIGURE S2 The relative intensities of 18 endogenous biomarkers in control, model and XSHG groups. ($\overline{X} \pm SD$, n = 10) $^{\#}P < 0.05$, $^{\#\#}P < 0.01$ compared to control group; while $^{*}P < 0.05$, $^{**}P < 0.01$ compared to model group.

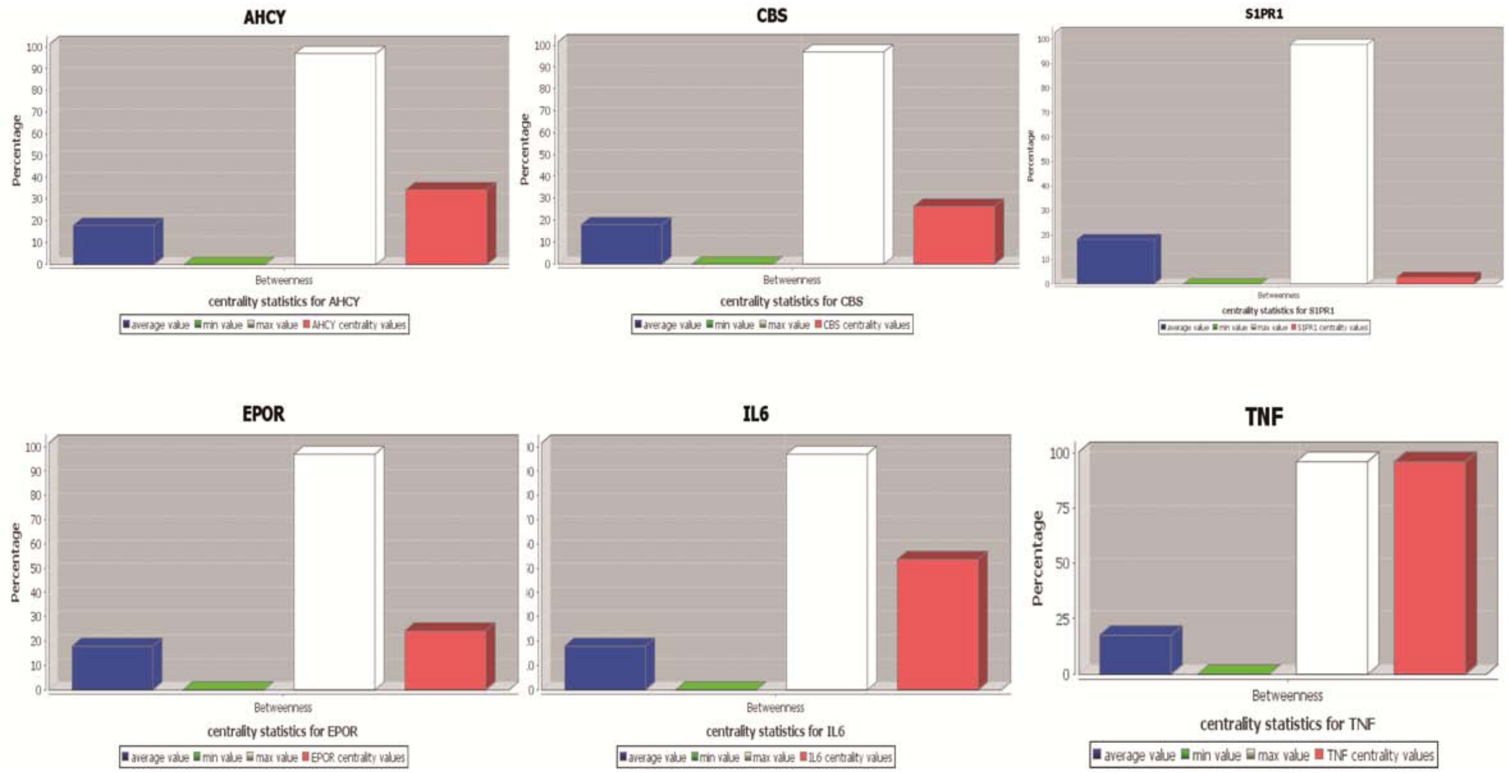

FIGURE S3 The correlation plots of the degree and betweenness centrality for targets AHCY, CBS, S1PR1, EPOR, IL6 and TNF.
